# A Precise and Stable Space-Based Time System for Navigation in Smart Cities

**DOI:** 10.3390/s24020480

**Published:** 2024-01-12

**Authors:** Shaoqian Li, Baojun Lin, Rui Li, Xiaogong Hu, Richang Dong

**Affiliations:** 1 Aerospace Information Research Institute, Chinese Academy of Sciences, Beijing 101408, China; lisq@microsate.com; 2 University of Chinese Academy of Sciences, Beijing 101408, China; lir@microsate.com; 3 Innovation Academy for Microsatellites, Chinese Academy of Sciences, Beijing 101408, China; dongrc@microsate.com; 4 Shanghai Engineering Center for Microsatellites, Shanghai 201203, China; 5 Aerospace Information Research Institute, University of Chinese Academy of Sciences, Innovation Academy for Microsatellites, Shanghai Engineering Center for Microsatellites, ShanghaiTech University, Shanghai 201210, China; 6 Shanghai Astronomical Observatory, Chinese Academy of Sciences, Shanghai 200030, China; hxg@shao.ac.cn

**Keywords:** satellite navigation system, smart city, space-based time system, inter-satellite link

## Abstract

The high-accuracy and high-stability space-based time system is necessary for satellite navigation systems to achieve high quality of service (QoS) on navigation and positioning in smart city applications. This paper proposes a precise and high-stability space-based time system established under the autonomous time scale of navigation satellites. The generation, maintenance, and transfer of high-precision space-based time references are researched. A centralized time comparison method based on the ALGOS algorithm conducts the two-way time comparison of the inter-satellite link. Specifically, using the relative clock difference observations of all links between satellites for a certain period of time, the clock difference, clock speed, and clock drift parameters of n−1 stars in a constellation of *n* stars relative to the same reference can be estimated simultaneously. Simulations are conducted on real collected data from the Beidou navigation systems when providing services to smart cities around the world. The simulation results show the high accuracy and stability of the proposed space-based time system under the autonomous time scale reference. Moreover, the clock offset monitoring arc coverage is much higher than the satellite clock offset obtained by the direct observation of the satellite and the anchor station. It proves the efficiency of the proposed space-based time system to be used for satellite clock offset modeling and prediction.

## 1. Introduction

The satellite navigation system focuses on the needs of national security and economic and social development [1,2]. It is an important national infrastructure that provides all-weather, all-day, high-precision positioning, navigation, and timing services for global users, especially in supporting smart city applications such as transportation, route planning, and self-driving cars [3,4]. Efficient satellite–terrestrial networks have been researched and built to support these applications [5,6,7,8]. Additionally, the satellite navigation system should also be supported by a high-accuracy and high-stability space-based time system to achieve high quality of service (QoS) on navigation and positioning in the above smart city applications, which is the main research target in this paper.

The core component to build a space-based time system for navigation constellations is the space-borne atomic clock [9,10,11]. The navigation satellites widely use rubidium clocks, cesium clocks, and passive hydrogen clocks as onboard atomic clocks. After decades of development, the performance of various space-borne atomic clocks has been greatly improved. The short-term stability of rubidium clocks is better than $1\times 10^{-12}/$ s, and the long-term stability is better than $1\times 10^{-14}/$ d. The long-term stability of the high-precision passive hydrogen clock is even better than $5\times 10^{-15}/$ d [9,12], which is equivalent to that of the GPS-3 space-borne pulsed light-pumped cesium clock [13]. The performance of new space-borne atomic clocks such as microwave clocks and integrating sphere cold atomic clocks is also improving [14,15,16].

An accurate time measurement system is key to determining the target position and providing precise positioning services for the navigation satellite system. The nowadays commonly used space-based time offset measurement methods, including the satellite-ground two-way time comparison method (TWTT) [17] and the orbit determination and time synchronization method (ODTS) [18,19], still have room for improvement in terms of precision. Therefore, this paper mainly focuses on the research of a space-based time system established under the autonomous time scale of navigation satellites. Specifically, it researches the generation, maintenance, and transfer of high-precision space-based time references. First of all, this paper proposes a scheme establishing a space-based time system for navigation constellations. The establishment follows the inter-satellite link two-way ranging, ranging information exchange, and data pre-processing to obtain the time comparison data of the entire network, and we use the time comparison data to obtain the space-based time with an integrated atomic time algorithm. The space-based integrated atomic time algorithm is realized based on the ALGOS algorithm [20].

For the evaluation of the stability of atomic clocks in orbit, the current satellite–ground time comparison method and the precise orbit determination and time synchronization method are compared, and a centralized time comparison method based on the two-way time comparison of the inter-satellite link is proposed. Using the relative clock difference observations of all links between satellites for a certain period of time, the clock difference, clock speed, and clock drift parameters of n−1 stars in a constellation of *n* stars relative to the same reference can be estimated simultaneously.

Simulations are conducted on real collected data from the Beidou navigation systems when providing services to smart cities around the world. The accuracy and stability of the proposed space-based time system are evaluated. The results show that the satellite clock offset has higher short-term and long-term prediction accuracy and stability under the autonomous time scale reference. Specifically, the residual error of the hydrogen clock offset prediction is decreased from 0.47 ns to 0.20 ns, and the residual error of the rubidium clock offset prediction is decreased from 0.50 ns to 0.38 ns. Based on the KA measurement data of the 18 MEO + 1 GEO satellites and an anchor station in orbit, an accuracy analysis is carried out. The analysis results show that for MEO satellites, the accuracy of the satellite clock offset fitting residual obtained by the centralized clock offset estimation parameter sequence is higher than that of the satellite–ground inter-satellite joint method, which has a 50% accuracy increment. The clock offset monitoring arc coverage is much higher than the satellite clock offset obtained by the direct observation of the satellite and the anchor station. Thus, our proposed method can be used for future satellite clock offset modeling and centralized clock offset prediction accuracy assessment.

## 2. Related Work

In practice, the time difference x(t) , frequency difference y(t) , and clock reading h(t) cannot be directly observed. The time calculation method according to the clock offset should be carefully researched. In this section, we survey two commonly used space-based time offset measurement methods, which are also compared with our proposed method in Section 5.

### 2.1. Space-Based Time Offset Measurement Methods

Two commonly used space-based time offset measurement methods are the satellite–ground two-way time comparison method (TWTT) and the orbit determination and time synchronization method (ODTS).

#### Satellite–Ground Two-Way Time Comparison Method

The TWTT method adopted the working mode of mutual transmission and reception. The ground station *A* transmitted the ranging signal to satellites at the ground time $T_{0A}$ . The signal was received by the satellite at the clock reading time $T_{s}$ , and the observation data were sent to the ground station. At the same time, the satellite transmitted the ranging signal at $T_{0}S$ time and was received by the ground station at the ground clock time $T_{A}$ . At this time, the uplink and downlink pseudo-ranges measured by the satellite and the ground [17] were

(1) $$\begin{array}{c} \rho_{S}^{\prime }=C\tau_{S}^{\prime }=C\left(T_{S}-T_{0}^{A}\right)=C\Delta T_{SA}+\rho_{S}\\ \rho_{A}^{\prime }=C\tau_{A}^{\prime }=C\left(T_{A}-T_{0}^{S}\right)=C\Delta T_{SA}+\rho_{A}. \end{array}$$

In this formula, *C* is the speed of light; $\Delta T_{SA}$ is the difference between the satellite clock and the ground clock; $\rho_{S}^{\prime }$ is the L-band uplink pseudo-range measured by the receiving equipment on the satellite; $\rho_{S}$ is the distance from the satellite to the ground station at $T_{s}$ time; $\rho_{A}^{\prime }$ is the L-band downlink pseudo-range measured by the ground receiving equipment; $\rho_{A}$ is the distance from the satellite to the ground station at time $T_{A}$ . The upper and lower formulas can be subtracted to yield

(2) $$\Delta T_{SA}=\frac{1}{2C}\left(\rho_{A}^{\prime }-\rho_{S}^{\prime }\right)+\frac{1}{2C}\left(\rho_{S}-\rho_{A}\right).$$

The difference between the satellite clock and the ground clock can therefore be obtained.

### 2.2. Orbit Determination and Time Synchronization Method

The precise ODTS use GNSS observation data to solve the precise orbit and clock offset of navigation satellites. This method generally uses ionospheric combination observations, and the error equation of its phase and pseudo-range observations can be, respectively, expressed as [18,19]

(3) $$v_{k,\Phi }^{j}\left(i\right)=\Delta t_{k}\left(i\right)-\Delta t^{j}\left(i\right)+\rho_{k}^{j}\left(i\right)/C+\delta \rho_{k,trop}^{j}\left(i\right)/C+\lambda \cdot N_{K}^{j}/C+\varepsilon_{k,\Phi }^{j}\left(i\right)-\lambda \cdot \Phi_{k}^{j}\left(i\right)/C,$$

(4) $$v_{k,p}^{j}\left(i\right)=\Delta t_{k}\left(i\right)-\Delta t^{j}\left(i\right)+\rho_{k}^{j}\left(i\right)/C+\delta \rho_{k,trop}^{j}\left(i\right)/C+\varepsilon_{k,p}^{j}\left(i\right)-P_{k}^{j}\left(i\right)/C,$$

where *j* is the satellite number; *k* is the station number; *i* is the corresponding observation epoch; $\Delta t_{k}\left(i\right)$ represents the receiver clock offset; $\delta \rho_{k,trop}^{j}\left(i\right)$ is the effect of tropospheric delay; $\Delta t^{j}\left(i\right)$ is the satellite clock offset; $\varepsilon_{k,\Phi }^{j}\left(i\right)$ and $\varepsilon_{k,p}^{j}\left(i\right)$ are error influences from multi-path and observation noise; $P_{k}^{j}\left(i\right)$ and $\Phi_{k}^{j}\left(i\right)$ are the combined observations of the corresponding satellite, station, and epoch; *L* is the corresponding wavelength; $\rho_{k}^{j}$ is the geometric distance between the satellite position and the receiver position at the moment of signal transmission [21].

## 3. Atomic Clocks and Time Scale Metrics

An atomic clock serves as a precise frequency source, producing a sinusoidal signal with a stable frequency. The output signal of each atomic clock, treated as a time scale, undergoes disturbances—both deterministic and random—resulting in phase and frequency deviations, thereby affecting its stability. This section introduces the output signal model of atomic clocks and two key performance metrics for the assessment of time scales.

### 3.1. Atomic Clock Output Signal Model

The ideal output of an atomic clock is expressed as

(5) $$u\left(t\right)=U_{0}\sin\left(2\pi v_{0}t\right),$$

where $U_{0}$ is the amplitude, and $v_{0}$ is the atomic clock frequency. This can be rewritten as

(6) $$u\left(t\right)=U_{0}\sin\left(2\pi v_{0}h_{0}\left(t\right)\right),$$

with $h_{0}\left(t\right)$ representing the ideal clock reading. In practical applications, random disturbances affect the amplitude and phase, resulting in an output signal approximation [22]:

(7) $$u\left(t\right)=\left[U_{0}+\varepsilon \left(t\right)\right]\sin\left(2\pi v_{0}h_{0}\left(t\right)+\varphi \left(t\right)\right),$$

where ε(t) is the amplitude disturbance, and φ(t) is the phase disturbance. This can be further expressed as

(8) $$u\left(t\right)=\left[U_{0}+\varepsilon \left(t\right)\right]\sin\left(2\pi v_{0}h\left(t\right)\right),$$

where $h\left(t\right)=h_{0}\left(t\right)+x\left(t\right)$ , and x(t) is the time deviation from the ideal reading.

For practical high-performance atomic clocks, amplitude perturbation is usually negligible, but phase perturbation is not. This phase disturbance introduces a frequency deviation:

(9) $$v\left(t\right)=v_{0}+\frac{1}{2\pi }\frac{d\varphi }{dt},$$

leading to the definition of frequency stability y(t) :

(10) $$y\left(t\right)=\frac{v\left(t\right)-v_{0}}{v_{0}}=\frac{1}{2\pi v_{0}}\frac{d\varphi (t)}{dt}=\frac{dx(t)}{dt}.$$

In practice, x(t) , y(t) , and the clock reading h(t) are not directly observed. The observed quantity is the clock offset $x_{ij}\left(t\right)$ between two clocks *i* and *j*:

(11) $$x_{ij}\left(t\right)=h_{i}\left(t\right)-h_{j}\left(t\right).$$

This can be expressed as

(12) $$x_{ij}\left(t\right)=x_{i}\left(t\right)-x_{j}\left(t\right).$$

To assess the performance of atomic clocks, the typical method involves measuring the clock offset between different atomic clocks, representing the difference in the time deviation of their output signals.

### 3.2. Performance Metrics for Time Scale

Evaluation metrics for atomic clocks include accuracy and stability.

#### 3.2.1. Accuracy

The frequency accuracy *A* of a time scale measures the correspondence between a unit second defined by the time scale and the one in the international system of units. It is defined as

(13) $$A=\frac{f-f_{0}}{f_{0}},$$

where *f* is the actual frequency of the measured frequency standard, and $f_{0}$ is its nominal frequency. Accuracy is crucial in comparing local atomic time with international atomic time.

#### 3.2.2. Stability

The stability of a time scale is its ability to maintain a constant scale interval. It quantifies how the frequency value changes over time. The Allan variance, proposed by D.W. Allan, is commonly used for this purpose. For phase data, it is expressed as [23]

(14) $$\sigma_{y}^{2}\left(\tau \right)=\frac{1}{2\left(N-2\right)\tau^{2}}\sum_{i=1}^{N-2}{\left[x\left(i+2\right)-2x\left(i+1\right)+x\left(i\right)\right]}^{2},$$

and for frequency data as

(15) $$\sigma_{y}^{2}\left(\tau \right)=\frac{1}{2(M-1)}\sum_{i=1}^{M-1}{\left[y\left(i+1\right)-y\left(i\right)\right]}^{2},$$

where τ is the smoothing time, often an integer multiple of the measurement time interval.

Hadamard variance, based on the secondary frequency deviation for frequency data and cubic deviation for phase data, helps to remove linear drift. For frequency data,

(16) $$H\sigma_{y}^{2}\left(\tau^{\prime }\right)=\frac{1}{6(M-2)}\sum_{i=1}^{M-2}{\left[y_{i+2}-y_{i+1}+y_{i}\right]}^{2},$$

and, for phase data,

(17) $$H\sigma_{y}^{2}\left(\tau^{\prime }\right)=\frac{1}{6\tau^{2}\left(N-3m\right)}\sum_{i=1}^{N-3}{\left[x_{i+3}-3x_{i+2}+3x_{i+1}-x_{i}\right]}^{2}.$$

Clock offset data can be directly obtained in observations, and the stability of one time scale is always relative to another. The three-cornered hat method is commonly employed, providing stability relationships among three time scales [24]. If the stability of a reference time scale is significantly better than the measured time scale, the measured stability can be considered as that of the measured time scale. If their stabilities are equivalent, the three-cornered hat method equations can be solved to obtain the stability of each time scale.

## 4. Design of the Space-Based Time System for Navigation Constellations

The space-based time system is constructed and maintained by a constellation clock group in the navigation constellation, which includes a configured high-performance atomic clock, inter-satellite measurement, and data transmission links. The middle- and high-orbit laser and microwave inter-satellite links are responsible for broadcasting the satellite inter-clock offset parameters, achieving time synchronization within the constellation.

Currently, the most widely used on-board time synchronization and autonomous orbit determination method is the distributed Kalman filter algorithm. However, it leads to a rapid increase in the time difference between the constellation and the ground, resulting not only in the decreased accuracy of the timing service but also indirectly affecting the accuracy of autonomous orbit determination, making it difficult to exceed 60 days. Two main reasons contribute to this problem.

1. The constellation timekeeping function is not designed, making it challenging to maintain constellation time accuracy for an extended period.
2. As the autonomous operation time of satellites approaches 60 days, the time scale error of constellation maintenance becomes less able to meet the needs of autonomous orbit determination, leading to a rapid deterioration in orbit determination precision.

Therefore, research on the timekeeping function of the navigation constellation is necessary. Considering the advantages of the timekeeping accuracy of the constellation clock group, the time–frequency performance of the constellation during autonomous operation should meet the quality of service (QoS) requirements of autonomous orbit determination and timing services.

To establish and maintain the high-precision space-based time system of the navigation system, this paper proposes the following:

1. Satellite clock stability evaluation technology based on inter-satellite link measurement;
2. The establishment of the satellite–ground joint integrated atomic time;
3. Space-based integrated atomic time that can support special circumstances based on the ALGOS algorithm.

This comprehensive space-based time system can provide a higher-stability frequency reference and system time for the satellite navigation system, thereby further increasing the QoS in smart city applications.

### 4.1. Time Scale Model

The clock source is a clock group composed of multiple hydrogen atomic clocks and cesium atomic clocks. The atomic clocks on each satellite serve as independent time references. For a clock group consisting of *N* atomic clocks, the time observation equation of any atomic clock *i* can be written in the form of the following formula, thus obtaining *N* time references:

(18) $$T_{i}=x_{0}+y_{0}t+\frac{1}{2}Dt^{2}+\varepsilon_{x}\left(t\right),$$

where $T_{i}$ is the time deviation of the atomic clock, $x_{0}$ is the initial time deviation, $y_{0}$ is the initial frequency deviation, *D* is the linear drift rate of the atomic clock, and $\varepsilon_{x}\left(t\right)$ corresponds to the random variable of the time deviation, i.e., the noise of the atomic clock. Here, we use a quadratic polynomial model for clock error prediction, taking into account the physical characteristics of the satellite clock error, frequency accuracy, frequency drift rate, and frequency stability, which are widely used clock error extrapolation methods in broadcast ephemeris. The clock error characteristic data of the satellite are constants fitted based on the on-orbit measurement results. These constants can be used to predict the satellite clock within a certain period of time.

The purpose of the time scale algorithm design is to optimize the stability of the time scale, so the atomic time algorithm is used. The weighted average of all atomic clocks can finally lead to higher reliability, stability, and accuracy of the final comprehensive atomic time scale than any atomic clock.

### 4.2. Overview of the Space-Based Time System Establishment

The space-based time system needs to support the dynamic access of space and Earth time and frequency resources. It should have strong flexibility and expansibility. The newly accessed atomic clock can instantly work, and the whole system can remain stable when any clock is offline. Additionally, this system should support the access of not only the atomic clock resources inside the navigation constellation but also the timekeeping clock of the space and the ground outside the navigation constellation. This means that the system can realize space–ground integrated timekeeping.

**Figure 1:**
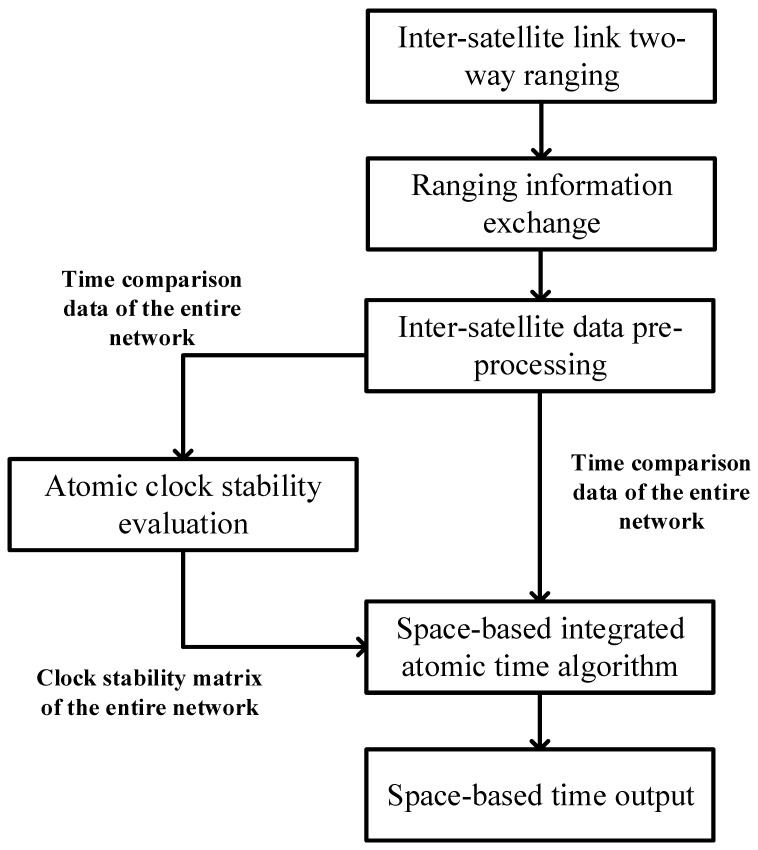
The space-based time system establishment process.

The process of the proposed space-based time system is shown in Figure 1. The construction of this space-based time system follows several steps.

1. Preprocess the inter-satellite two-way ranging data to obtain the time comparison data of the entire network. Taking the ranging between two satellites as an example, the data preprocessing steps include ionospheric delay correction, relativity correction, outlier elimination, and epoch correction. The pseudo-range observation equation for inter-satellite two-way ranging is established, and the whole network time comparison data are accordingly calculated.
2. Acquire the stability matrix of the entire constellation from the time comparison data. The comprehensive frequency stability of two clocks in each group is obtained using the distributed computing method. Then, the frequency stability of the atomic clock of each star is acquired by the “triangular hat” method.
3. Calculate the atomic clock weight factor. The average atomic time scale reflects the noise suppression of the integrated clock, and an atomic clock with good stability has a great weight. Combined with on-orbit data, the atomic clock weight factor is calculated under the comprehensive analysis of the number of clocks, calculation cycle, weight variance interval, and maximum weight settings.
4. Output the space-based time. Comparing the advantages and disadvantages of the weighted average algorithm (including the ALGOS algorithm and at1 algorithm), Kalman filter algorithm, wavelet decomposition algorithm, etc., a timekeeping algorithm suitable for establishing space-based time based on the ALGOS algorithm is proposed.

### 4.3. Centralized Two-Way Time Comparison Method Based on Inter-Satellite Link

For domestic satellites, the TWTT method is used to obtain the satellite–ground clock difference of the domestic arc segment. However, for overseas satellites, the effective tracking of MEO satellites by the regional tracking network is less than 40% of the entire arc segment. When the satellite moves overseas, the observation data are missing, and the broadcast ephemeris and clock error parameters cannot be updated in time. To solve this problem, the satellite–ground inter-satellite joint method is adopted. This method takes domestic satellites as relay nodes. It leverages the satellite–ground clock differences of domestic satellites and the inter-satellite relative clock differences between domestic and foreign satellites to obtain the clock error of the satellite’s outer arc segment relative to BDT by one-hop communication. Finally, the domestic and external clock differences of each satellite are fitted to the satellite clock parameters to obtain the satellite broadcast clock parameters.

The fundamental observation equation of the two-way inter-satellite link is expressed as follows [25,26]:

(19) $$\begin{array}{cc} \rho_{AB}\left(t_{1}\right) & =\left|{\vec{R}}_{B}\left(t_{1}\right)-{\vec{R}}_{A}\left(t_{1}-\Delta t_{1}\right)\right|\\ \; & \;+c^{*}\left(clk_{B}\left(t_{1}\right)-clk_{A}\left(t_{1}\right)+\tau_{A}^{Send}+\tau_{B}^{Rcv}\right)+\Delta \rho_{cor}^{AB}\\ \rho_{BA}\left(t_{2}\right) & =\left|{\vec{R}}_{B}\left(t_{2}\right)-{\vec{R}}_{A}\left(t_{2}-\Delta t_{2}\right)\right|\\ \; & \;+c^{*}\left(clk_{A}\left(t_{2}\right)-clk_{B}\left(t_{2}\right)+\tau_{A}^{Rcv}+\tau_{B}^{Send}\right)+\Delta \rho_{cor}^{BA}. \end{array}$$

In the above equations, $\rho_{AB}\left(t_{1}\right)$ and $\rho_{BA}\left(t_{2}\right)$ represent the pseudo-range observations received by satellites A and B at times $t_{1}$ and $t_{2}$ . Other parameters are defined as follows: ${\vec{R}}_{A}$ and ${\vec{R}}_{B}$ are the three-dimensional position vectors of satellites A and B; $clk_{A}$ and $clk_{B}$ represent the clock offset of satellites A and B; *c* is the speed of light; Δt is the light travel time; $\tau_{A}^{Send}$ and $\tau_{B}^{Send}$ are the transmission delays of satellites A and B; $\tau_{A}^{Rcv}$ and $\tau_{B}^{Rcv}$ are the receiving delays; $\Delta \rho_{cor}^{AB}$ and $\Delta \rho_{cor}^{BA}$ are error correction terms.

To obtain two-way clock offset or two-way distance observations, it is necessary to unify the one-way observations at the same time. The uniform equations are given by [26]

(20) $$\begin{array}{cc} \rho_{AB}\left(t_{0}\right) & =\rho_{AB}\left(t_{1}\right)+d\rho_{AB}\\ \rho_{BA}\left(t_{0}\right) & =\rho_{BA}\left(t_{2}\right)+d\rho_{BA}, \end{array}$$

where $d\rho_{AB}$ and $d\rho_{BA}$ represent the uniform correction amount from the observation time to the unified time.

The uniform correction amounts can be calculated using the satellite prediction orbit and clock offset parameters. They are defined by

(21) $$\begin{array}{cc} d\rho_{AB} & =\left|{\vec{R}}_{B}\left(t_{0}\right)-{\vec{R}}_{A}\left(t_{0}\right)\right|\\ \; & \;-\left|{\vec{R}}_{B}\left(t_{1}\right)-{\vec{R}}_{A}\left(t_{1}-\Delta t_{1}\right)\right|\\ \; & \;+c^{*}\left(clk_{B}\left(t_{1}\right)-clk_{A}\left(t_{1}\right)\right)\\ \; & \;-c^{*}\left(clk_{B}\left(t_{0}\right)-clk_{A}\left(t_{0}\right)\right), \end{array}$$

(22) $$\begin{array}{cc} d\rho_{BA} & =\left|{\vec{R}}_{B}\left(t_{0}\right)-{\vec{R}}_{A}\left(t_{0}\right)\right|\\ \; & \;-\left|{\vec{R}}_{B}\left(t_{2}\right)-{\vec{R}}_{A}\left(t_{2}-\Delta t_{2}\right)\right|\\ \; & \;+c^{*}\left(clk_{A}\left(t_{2}\right)-clk_{B}\left(t_{2}\right)\right)\\ \; & \;-c^{*}\left(clk_{A}\left(t_{0}\right)-clk_{B}\left(t_{0}\right)\right). \end{array}$$

By subtracting the two-way observations at the unified time, the satellite orbit information can be eliminated, and the inter-satellite two-way clock offset observation equation can be obtained:

(23) $$\begin{array}{cc} \frac{\rho_{AB}\left(t_{0}\right)-\rho_{BA}\left(t_{0}\right)}{2} & =c\cdot \left[clk_{B}\left(t_{0}\right)-clk_{A}\left(t_{0}\right)\right]\\ \; & \;+c\cdot \frac{\tau_{A}^{Send}-\tau_{A}^{Rcv}}{2}\\ \; & \;-c\cdot \frac{\tau_{B}^{Send}-\tau_{B}^{Rcv}}{2}+\frac{\Delta \rho_{cor}^{AB}-\Delta \rho_{cor}^{BA}}{2}. \end{array}$$

For 1-min uniform time intervals, redundant two-way observations can be obtained, facilitating “one satellite–multiple links” at a uniform moment. The simplified form of Equation (23) is

(24) $$\frac{\rho_{AB}^{\prime }\left(t_{0}\right)-\rho_{BA}^{\prime }\left(t_{0}\right)}{2}=c\times \left(CLK_{BA}\right),$$

where $\rho_{AB}^{\prime }\left(t_{0}\right)$ and $\rho_{BA}^{\prime }\left(t_{0}\right)$ are defined as

(25) $$\begin{array}{cc} \rho_{AB}^{\prime }\left(t_{0}\right) & =\rho_{AB}\left(t_{0}\right)-\Delta \rho_{cor}^{AB},\\ \rho_{BA}^{\prime }\left(t_{0}\right) & =\rho_{BA}\left(t_{0}\right)-\Delta \rho_{cor}^{BA}. \end{array}$$

The right side of the equation is given by

(26) $$CLK_{BA}\left(t_{0}\right)=CLK_{BA}^{0}\left(t_{0}\right)-\left[\frac{\tau_{B}^{Send}-\tau_{B}^{Rcv}}{2}-\frac{\tau_{A}^{Send}-\tau_{A}^{Rcv}}{2}\right].$$

The first term of Equation (26) is the relative clock offset of the two satellites:

(27) $$CLK_{BA}^{0}\left(t_{0}\right)=clk_{B}\left(t_{0}\right)-clk_{A}\left(t_{0}\right).$$

The second term of Equation (26) is the delay difference of the Ka combination, which can be considered as a constant within a certain period and calibrated in advance. If a star is selected as the reference star (*q* subscript), such that $clk_{q}\left(t\right)-\frac{\tau_{q}^{Send}-\tau_{q}^{Rcv}}{2}=0$ , then $CLK_{qq}=0$ . The relative clock offset observation equation of each star relative to the reference star can be obtained:

(28) $$\frac{\rho_{AB}^{\prime }\left(t_{0}\right)-\rho_{BA}^{\prime }\left(t_{0}\right)}{2c}=CLK_{Bq}-CLK_{Aq}.$$

For the Ka inter-satellite clock offset observations of the *i*-th and *j*-th satellites in a constellation of *n* stars,

(29) $$clk_{ij}\left(t\right)=\left(A_{0}^{I}+A_{1}^{i}\left(t-t_{0}\right)+A_{2}^{i}{\left(t-t_{0}\right)}^{2}\right)-\left(A_{0}^{J}+A_{1}^{j}\left(t-t_{0}\right)+A_{2}^{j}{\left(t-t_{0}\right)}^{2}\right)+\varepsilon_{ij}.$$

In the above formula, $A_{0}$ represents the clock error, $A_{1}$ represents the clock drift, and $A_{2}$ represents the clock drift rate. By taking the relative clock offset observations of all links between satellites for a certain period, the clock offset, clock velocity, and clock drift parameters of n−1 stars in a constellation of *n* stars can be estimated. The transmission and receiving delay on satellites only affect the clock offset parameters, not the clock velocity and clock drift parameters. If the constellation contains only satellites, time synchronization among all satellites is achieved.

The reference for the broadcast clock offset parameters from the navigation satellite system is the system time. By adding the relative clock offset observations between the anchor station and the satellite based on the *n*-satellite constellation, and selecting the anchor station as the reference, the clock offset, clock velocity, and clock drift parameters can be directly broadcast.

### 4.4. Space-Based Integrated Atomic Time Algorithm Based on ALGOS Algorithm

From Equation (28), it is evident that the inter-satellite clock offset reflects the relative clock offset of two satellites, and its noise encompasses the noise of the two clocks. The ALGOS method is employed to construct the inter-satellite integrated atomic time, enabling a more accurate evaluation of the space-borne atomic clock noise.

The ALGOS algorithm, initially utilized by the International Bureau for Weights and Measures (BIPM) [20], generates the international reference Coordinated Universal Time (UTC) on a monthly basis. The UTC calculation using ALGOS involves three steps:

1. The Echelle Atomique Libre (EAL) is calculated as a weighted average of 350 free-running atomic clocks, optimizing the long-term frequency stability through a specially designed weighting method;
2. The frequency of EAL maintains agreement with the International System of Units (SI) second, providing the International Atomic Time (TAI); the comparison between the EAL frequency and the primary frequency standards yields the steering correction;
3. Coordinated Universal Time (UTC) is finally calculated, incorporating leap seconds to maintain agreement with the time derived from the rotation of the Earth.

To implement the ALGOS method, determining the weights of different space-borne atomic clocks is necessary. Using a star with high stability as a reference (as introduced in Section 4.3), the clock offset of each satellite relative to a certain satellite is estimated. The acquired clock offset sequence is then regressed long-term to deduce the clock drift and speed. The top-N space-borne atomic clocks with the highest stability are selected, and comprehensive atomic time is constructed in an equal-weighted manner:

(30) $$TA1\left(t_{0}\right)-clk_{A}\left(t_{0}\right)=\frac{\sum_{i=1}^{N}CLK_{iA}^{0}\left(t_{0}\right)}{N},$$

where *N* is the number of satellites when constructing the synthetic atom, and *i* is the index of the satellite. Although the absolute value of the preliminary integrated atomic time $TA1(t_{0})$ cannot be obtained, the clock offset of the reference star relative to the integrated atomic time can be obtained by Equation (30). Subtracting Equations (27) and (30), the clock offset of each star relative to the integrated atomic time can be obtained as follows:

(31) $$TA1\left(t_{0}\right)-clk_{B}\left(t_{0}\right)=TA1\left(t_{0}\right)-clk_{A}\left(t_{0}\right)-\left(clk_{B}\left(t_{0}\right)-clk_{A}\left(t_{0}\right)\right).$$

Finally, the space-based atomic time is output as

(32) $$TA1\left(t_{0}\right)-clk_{A}\left(t_{0}\right)=\frac{\sum_{i=1}^{N}\omega_{i}CLK_{iA}^{0}\left(t_{0}\right)}{N},$$

where $\omega_{i}\left(t\right)$ is determined based on the Allan variance of the space-borne atomic clock offset.

## 5. Evaluation Results

The autonomous time scale is a time scale established based on atomic clocks carried by multiple satellites and does not rely on the ground. It is established using seven hydrogen clocks, with the clock offset of all satellites obtained relative to this autonomous time scale. The evaluation data are collected from the Beidou satellite navigation system while providing services globally.

In our simulations, the Beidou-3 satellite link establishment is conducted in 1-min units. This approach allows the connection of the entire constellation based on the relative clock difference data of the inter-satellite links within 1 min, ensuring the feasibility of the proposed clock error parameter estimation method. The ground station uses a clock group to keep time and is traceable to China’s local atomic time TA (NTSC). Its stability is 8.3 × $10^{16}$ (5 days) and 4.7 × $10^{16}$ (30 days).

The performance of the generated space-based time scale is evaluated through the comparison of satellite clock offset fitting residuals and prediction errors. The stability of the space-based time system is also assessed. Towards the end of this section, the accuracy of the centralized two-way time comparison method based on the inter-satellite link is evaluated and compared with that of integration- and anchor-based methods.

### 5.1. Satellite Clock Offset Fitting Residuals

**Figure 2:**
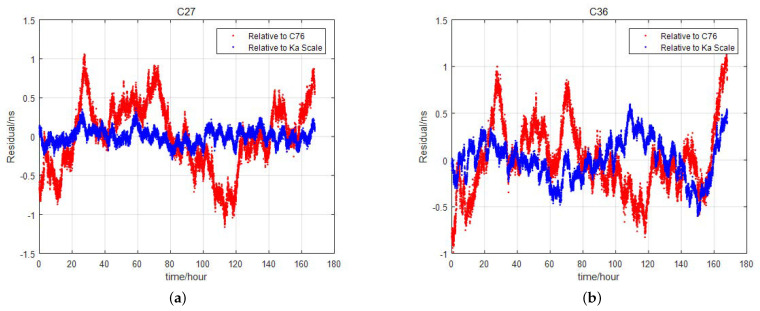
Evaluation of clock offset fitting residuals for two satellites relative to anchor station (red) and to autonomous time scale (blue). (**a**) Fitting residual evaluation on satellite C27. (**b**) Fitting residual evaluation on satellite C36.

The satellite clock offset fitting residual is calculated through second-order polynomial fitting relative to the clock offset of the autonomous time scale. This residual is compared with the fitting residual error of the satellite relative to the clock offset of the anchor station. The comparison results for some satellites are shown in Figure 2.

Results for 7 days are shown in Figure 2, where red represents the residual error of the clock offset fitting of the satellite relative to the anchor station, and blue represents the residual error relative to the autonomous time scale. The C27 satellite is equipped with a hydrogen clock, while the C36 satellite has a rubidium clock. Different space-borne clocks exhibit improved accuracy in the clock offset relative to the autonomous time scale, with hydrogen clocks showing a more significant improvement.

Quantitatively, the Root-Mean-Square (RMS) statistics of the clock offset fitting residuals are shown in Table 1. The table indicates that when converting the reference from anchor station clocks to autonomous time scales, the 7-day clock offset residual error for hydrogen clocks decreases from 0.47 ns to 0.20 ns, and for rubidium clocks, it decreases from 0.50 ns to 0.38 ns.

### 5.2. Satellite Clock Offset Prediction Error

The primary goal of establishing a space-based time system is to serve users in various scenarios, such as smart cities. Therefore, it is crucial to evaluate the accuracy of the satellite broadcast clock parameters. The satellite clock offset prediction is traditionally calculated under the anchor station scale. However, this paper proposes to conduct the satellite clock offset prediction under the autonomous time scale. The evaluation strategy includes both short-term and long-term predictions.

In the short-term prediction, first-order polynomial fitting is applied to 2-h clock offset data to predict the next 2 h. The 2-h prediction error RMS is then analyzed. Conversely, the long-term prediction involves fitting on 48-h clock offset data. Hydrogen clocks and C34 use a first-order polynomial, while other rubidium clocks use a second-order polynomial. The 24-h clock offset data are further predicted, and the prediction error RMS is calculated accordingly. The short-term and long-term prediction accuracy of the satellite clock offset under the anchor station clock and the autonomous time scale are summarized in Table 2.

The statistical results of the prediction error show that by converting the time reference from the anchor station clock to the autonomous time scale, the 2-h prediction error RMS of the satellite clock is decreased from 0.20 ns to 0.15 ns. The 24-h prediction error RMS of the hydrogen clock is decreased from an average of 1.07 ns to 0.45 ns, and the RMS prediction error of the rubidium clock in the 24th h is decreased from an average of 1.98 ns to 1.40 ns. In summary, the satellite clock offset prediction demonstrates higher short-term and long-term accuracy under the autonomous time scale reference.

### 5.3. Space-Based Time System Stability Evaluation

**Figure 3:**
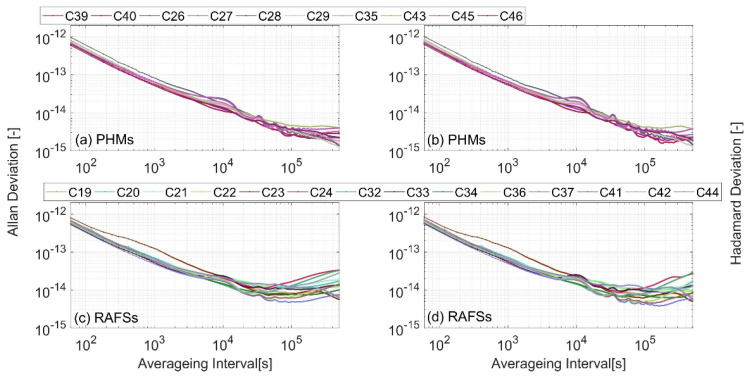
The Allan deviation and Hadamard deviation of space-based time system stability under anchor station reference (PHMs) and autonomous time scale reference (RAFSs).

The frequency stability of the space-based time system is evaluated based on the satellite clock offset under two benchmarks: an anchor station reference and autonomous time scale reference. The influence of clock drift is deducted in the evaluation. The Allan variance of the satellite clock offset under the anchor station clock reference is shown in Figure 3a, and the Allan variance of the satellite clock offset under the autonomous time scale reference is shown in Figure 3b.

Quantitatively, the statistics of satellite clock stability under the two benchmarks are shown in Table 3. Under the anchor station benchmark, the 10,000-s stability of hydrogen clocks and rubidium clocks is approximately $2.6-2.8\times 10^{-14}$ . The average daily stability of hydrogen clocks is $8.67\times 10^{-15}$ , and the average daily stability of rubidium clocks is $1.18\times 10^{-14}$ . Under the autonomous time scale benchmark, the 10,000-s stability of hydrogen clocks and rubidium clocks is about $1.8\times 10^{-14}$ . The average daily stability of hydrogen clocks is $3.59\times 10^{-15}$ , and the average daily stability of rubidium clocks is $8.51\times 10^{-15}$ .

Based on the above results, it is reasonable that the frequency stability of autonomous time scales is higher than that of anchored station clocks. Furthermore, the Allan variance of the anchor station clock is evaluated by the clock offset of the anchor station relative to the autonomous time scale, and the results are shown in Figure 3c. The 10,000-s stability of the anchored station clock estimated by this method is about $2.1\times 10^{-14}$ , and the day stability is about $7.6\times 10^{-15}$ . Smoothing the time to the day, the stability of the space-borne hydrogen clock and some rubidium clocks is better than that of the anchor station clock. This also explains the phenomenon that the Allan variance curves of satellite clocks are difficult to distinguish under the anchor station reference.

### 5.4. Accuracy Evaluation of the Proposed Centralized Two-Way Time Comparison Method Based on the Inter-Satellite Link

The Beidou-3 satellite takes 1 min as a unit to complete the link establishment of different inter-satellite links. In other words, the relative clock offset data of the inter-satellite link within 1 min can complete the connection of the entire constellation. Therefore, the centralized prediction of the clock offset parameters of all constellation satellites can be completed by using the relative clock offset of all Ka links within 1 min. Due to the short duration, the influence of satellite clock drift is negligible. To avoid over-parameterization, only the $A_{0}$ and $A_{1}$ parameters are estimated for each satellite every minute.

The relative clock offset parameters of all links within 1 min of the 18 MEO + 1 GEO + 1 anchor station are used to estimate the centralized clock offset parameters. The sliding window is 1 min, and the reference time is the starting time of each minute. Table 4 shows the link residual RMS value based on satellite statistics. It can be seen from the table that, according to satellite statistics, the maximum residual RMS of 1-min centralized clock offset estimation is 0.251 ns, the minimum is 0.081 ns, and the average is about 0.143 ns.

**Figure 4:**
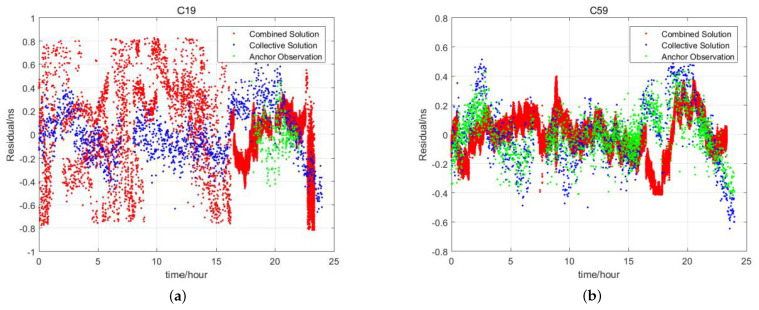
Comparison of fitting residuals of satellite clock offset obtained by different methods. (**a**) Fitting residual comparison on satellite C19 (MEO satellite). (**b**) Fitting residual comparison on satellite C59 (GEO satellite).

The evaluation takes the parameter $A_{0}$ sequence of each satellite as the satellite clock offset sequence sampled in 1 min and compares it with the satellite clock offset obtained by the satellite–ground inter-satellite joint method and anchor satellite method. The comparison is carried out by the second-order polynomial fitting. The fitting residuals are compared in Figure 4.

In Figure 4, C19 on the left is a medium-Earth-orbit (MEO) satellite, and C59 on the right is a geostationary (GEO) satellite. The red curve in the figure is the satellite clock offset fitting residual sequence obtained by the satellite–ground inter-satellite joint method, the blue curve is the satellite clock offset fitting residual sequence obtained by the centralized clock offset estimation method, and the green curve is the sequence obtained by direct observations between the satellite and the anchor station. The following conclusions can be drawn from the figure.

1. For MEO satellites, the satellite clock offset fitting residual accuracy obtained by the centralized clock offset estimation parameter sequence is higher than the one obtained by the satellite–ground inter-satellite joint method. The clock offset monitoring arc coverage is much higher than that of the direct observation between the satellite and anchor station.
2. For GEO satellites, the satellite clock offset obtained by the satellite–ground inter-satellite joint method is all L-band satellite–ground two-way time–frequency transfer clock offset. Since the GEO satellites are continuously visible, the three clock offset monitoring arc coverage is basically the same.
3. As for the noise level, the satellite clock offset noise of the L-band satellite–ground two-way time–frequency transmission is the smallest, followed by the satellite clock offset obtained by the direct observation of the anchor station, and the satellite clock offset obtained by the centralized clock offset estimation is slightly louder.

The fitting residual statistics of the above three clock offsets are shown in Table 5. Due to the particularity of GEO satellites, only 18 MEO satellites are averaged. It can be seen from the table that using the satellite–ground inter-satellite joint method, the mean value of the RMS of the 24-h clock offset fitting residual of the MEO satellite is 0.42 ns. The mean value of the 24-h satellite clock offset fitting residual RMS of the anchor station is 0.094 ns. The estimated 24-h satellite clock offset fitting residual RMS mean is 0.21 ns. The residual RMS of the clock offset fitting directly observed by the anchor station is small, which is mainly caused by the very short link establishment time between the MEO satellite and the anchor station.

To summarize, the satellite clock offset noise estimated by the centralized algorithm based on the inter-satellite link proposed in this paper is a more ideal method, since it has higher accuracy in satellite clock offset estimation and longer clock offset monitoring arc coverage.

### 5.5. Discussion

The purpose of establishing the navigation constellation space-based integrated atomic time is to obtain a stable and real-time space-based time benchmark. It has great value in improving the timekeeping accuracy of the navigation constellation and reducing its dependence on the ground.

The ALGOS algorithm adopted in this paper can provide higher stability. It assigns different weights to atomic clocks with different performance, where the weights can be estimated by the frequency stability of atomic clocks. However, this algorithm has only one stable weight for each clock; thus, it cannot take both short-term stability and long-term stability into consideration. The output time scale stability can only be optimized at a specified smoothing time point. The ALGOS algorithm is also a non-real-time algorithm and cannot generate a time reference in real time.

Conversely, the Kalman filter is based on a certain dynamic model, which is real-time and can predict the state of the system. For different signal models, different Kalman filters will be obtained. A Kalman filter is a vector signal processor that can handle all measurements in a clock group, including the time difference, frequency difference, and frequency drift of atomic clocks. To design a reasonable Kalman filter, the key lies in modeling the noise of the atomic clock reasonably and accurately estimating the noise variance in its driving noise covariance matrix, so as to eliminate the influence of colored noise on the Kalman filter.

In the future, our research on the space-based comprehensive time system will continue based on the Kalman filter algorithm and the ALGOS algorithm. Different atomic clocks have different noise models, and their corresponding covariance matrix calculation methods should also be adaptively improved, where we expect to obtain real-time, highly reliable, highly accurate space-based time.

## 6. Conclusions

As an important national infrastructure that provides all-weather, all-day, high-precision positioning, navigation, and timing services for global users, it is crucial to research a precise and stable navigation constellation space-based time system to better support smart city applications such as transportation, route planning, and self-driving cars. The purpose of establishing the space-based integrated atomic time of the navigation constellation is to obtain a stable and real-time space-based time reference, improve the system’s timekeeping accuracy, and reduce the dependence on the ground.

This paper proposes a space-based time system established under the autonomous time scale of navigation satellites. It consists of the establishment process, a centralized time comparison method based on the two-way time comparison of the inter-satellite link, the elimination of the magneto-frequency shift effect of space-borne atomic clocks, and the space-based integrated atomic time algorithm realized based on the ALGOS algorithm. As summarized in Table 6, simulations conducted on real collected Beidou navigation systems when providing services to smart cities around the world show the high accuracy and stability of the centralized estimation time system compared with the widely used anchor-based estimation. Moreover, the clock offset monitoring arc coverage is satisfied. Thus, the centralized estimation adopted in this paper has the potential to be used for satellite clock offset modeling and centralized clock offset prediction accuracy assessment for the next generation of navigation constellations.

## Figures and Tables

**Table 1:** RMS comparison of satellite clock offset fitting residuals under different references (unit: ns).

Satellite Number	Anchor Station Reference	Autonomous Time Scale Reference	Satellite Number	Anchor Station Reference	Autonomous Time Scale Reference
C25	0.61	0.33	C22	0.35	0.48
C26	0.40	0.14	C23	0.78	0.42
C27	0.43	0.10	C24	0.56	0.31
C28	0.40	0.13	C32	0.42	0.17
C29	0.38	0.10	C33	0.70	0.40
C30	0.43	0.16	C34	0.40	0.39
C59	0.62	0.43	C35	0.42	0.20
C20	0.82	0.58	C36	0.36	0.21
C21	0.62	0.56	C37	0.39	0.42
Average value of hydrogen clock	0.47	**0.20**	Average value of rubidium clock	0.50	**0.38**

**Table 2:** RMS values of satellite clock offset prediction errors under different references.

Satellite Number	Short-Term Prediction		Long-Term Prediction	
	Anchor Station Reference	Autonomous Time Scale Reference	Anchor Station Reference	Autonomous Time Scale Reference
C25	0.17	0.11	1.52	0.96
C26	0.21	0.13	0.89	0.24
C27	0.20	0.12	0.93	0.25
C28	0.23	0.17	0.87	0.25
C29	0.19	0.12	0.93	0.19
C30	0.23	0.17	0.89	0.56
C59	0.19	0.14	1.49	0.71
C20	0.21	0.16	2.31	1.79
C21	0.22	0.18	2.44	1.89
C22	0.20	0.16	2.18	2.25
C23	0.21	0.17	2.87	1.67
C24	0.24	0.18	2.14	1.10
C32	0.20	0.15	1.28	0.68
C33	0.20	0.16	1.78	1.60
C34	0.17	0.11	2.18	1.47
C35	0.21	0.15	1.94	0.86
C36	0.22	0.18	1.18	0.89
C37	0.19	0.15	1.45	1.16
Average Value of Hydrogen Clock	0.20	**0.14**	1.07	**0.45**
Average Value of Rubidium Clock	0.21	**0.16**	1.98	**1.40**

**Table 3:** The RMS values of satellite clock offset prediction error under different references.

Satellite Number	Anchor Station Reference		Autonomous Time Scale Reference	
	10,000-s Stability	Day Stability	10,000-s Stability	Day Stability
C25	$2.50\times 10^{-14}$	$9.92\times 10^{-15}$	$1.39\times 10^{-14}$	$6.52\times 10^{-15}$
C26	$2.52\times 10^{-14}$	$8.32\times 10^{-15}$	$1.48\times 10^{-14}$	$2.67\times 10^{-15}$
C27	$2.50\times 10^{-14}$	$7.83\times 10^{-15}$	$1.48\times 10^{-14}$	$1.93\times 10^{-15}$
C28	$2.96\times 10^{-14}$	$8.37\times 10^{-15}$	$2.30\times 10^{-14}$	$2.53\times 10^{-15}$
C29	$2.67\times 10^{-14}$	$7.80\times 10^{-15}$	$1.47\times 10^{-14}$	$1.95\times 10^{-15}$
C30	$3.12\times 10^{-14}$	$8.12\times 10^{-15}$	$2.20\times 10^{-14}$	$2.58\times 10^{-15}$
C59	$3.43\times 10^{-14}$	$1.03\times 10^{-14}$	$2.62\times 10^{-14}$	$6.91\times 10^{-15}$
C20	$2.62\times 10^{-14}$	$1.49\times 10^{-14}$	$1.94\times 10^{-14}$	$1.39\times 10^{-14}$
C21	$2.87\times 10^{-14}$	$1.75\times 10^{-14}$	$2.25\times 10^{-14}$	$1.42\times 10^{-14}$
C22	$2.59\times 10^{-14}$	$1.13\times 10^{-14}$	$1.80\times 10^{-14}$	$1.05\times 10^{-14}$
C23	$2.73\times 10^{-14}$	$1.41\times 10^{-14}$	$2.09\times 10^{-14}$	$7.87\times 10^{-15}$
C24	$3.08\times 10^{-14}$	$1.20\times 10^{-14}$	$2.14\times 10^{-14}$	$6.65\times 10^{-15}$
C32	$2.16\times 10^{-14}$	$6.93\times 10^{-15}$	$1.31\times 10^{-14}$	$4.22\times 10^{-15}$
C33	$2.74\times 10^{-14}$	$1.52\times 10^{-14}$	$2.08\times 10^{-14}$	$1.06\times 10^{-14}$
C34	$2.39\times 10^{-14}$	$1.13\times 10^{-14}$	$1.34\times 10^{-14}$	$8.54\times 10^{-15}$
C35	$2.74\times 10^{-14}$	$1.02\times 10^{-14}$	$1.79\times 10^{-14}$	$5.37\times 10^{-15}$
C36	$2.54\times 10^{-14}$	$7.79\times 10^{-15}$	$1.99\times 10^{-14}$	$5.29\times 10^{-15}$
C37	$2.47\times 10^{-14}$	$8.64\times 10^{-15}$	$1.47\times 10^{-14}$	$6.40\times 10^{-15}$
The average value of the hydrogen clock	$2.81\times 10^{-14}$	$8.67\times 10^{-15}$	$1.85\times 10^{-14}$	$3.59\times 10^{-15}$
The average value of the rubidium clock	$2.63\times 10^{-14}$	$1.18\times 10^{-14}$	$1.84\times 10^{-14}$	$8.51\times 10^{-15}$

**Table 4:** The residual statistics of 1-min centralized inter-satellite clock offset parameter estimation (unit: ns).

Satellite Number	Residual RMS	Satellite Number	Residual RMS
C19	0.12	C29	0.19
C20	0.11	C30	0.16
C21	0.16	C32	0.15
C22	0.13	C33	0.12
C23	0.22	C34	0.16
C24	0.10	C35	0.13
C25	0.19	C36	0.19
C26	0.25	C37	0.09
C27	0.15	C59	0.10
C28	0.16	Anchor station	0.08
Average	**0.14**		

**Table 5:** The RMS values of satellite clock offset prediction error through different methods.

Satellite Number	Satellite-Ground and Inter-Satellite Integration	Anchor Station Observation	Centralized Estimation
C19	0.23	0.16	0.22
C20	0.53	0.09	0.21
C21	0.51	0.07	0.28
C22	0.30	0.08	0.18
C23	0.67	0.08	0.21
C24	0.37	0.08	0.22
C25	0.35	0.12	0.24
C26	0.28	0.08	0.24
C27	0.33	0.12	0.19
C28	0.48	0.09	0.17
C29	0.36	0.09	0.17
C30	0.11	0.07	0.24
C32	0.49	0.09	0.19
C33	0.48	0.08	0.17
C34	0.49	0.13	0.17
C35	0.65	0.08	0.22
C36	0.49	0.09	0.19
C37	0.41	0.08	0.23
C59	0.13	0.15	0.22
The average value of MEO satellites	0.42	0.09	**0.21**

**Table 6:** A summary of the advantages of centralized estimation compared with the anchor-based method.

Method	Latency	Accuracy	Noise	Coverage	Stability
Anchor-based method	Short	Low	Low	Medium	Medium
Centralized estimation	**Short**	**High**	**Low**	**High**	**High**
